# Obstruction of ventriculoperitoneal shunt by air bubble: A case report

**DOI:** 10.1016/j.amsu.2022.104351

**Published:** 2022-08-24

**Authors:** Marouane Makhchoune, Michel Triffaux, Triantafyllos Bouras, Xavier Hamoir

**Affiliations:** a Neurosurgery Department, Hospital Center of Wallonie Picarde, Tournai, Belgium; b Radiology Department, Hospital Center of Wallonie Picarde, Tournai, Belgium

**Keywords:** Shunt failure, CT SCAN, Pneumocephalus, Hydrocephalus, Case report

## Abstract

Complications related to Ventriculoperitoneal shunt placement are common, and multiple. Among them blockage and infection. We report a case of 44 years old man admitted to our hospital after an obstruction of his ventriculo-peritoneal shunt by an air bubble that caused behavioral problems and confusion. The patient was operated twice, the last time the puncture point had to be changed. The follow up was marked by a clear clinical improvement. Shunt malfunction continues to be a common neurosurgical problem in patients with shunted hydrocephalus, often leading to frequent and sometimes lengthy hospital stays. This case illustrates the management of this rare situation causing air bubble shunt obstruction.

## Highlights

• Complications related to Ventriculoperitoneal shunt placement are common, and multiple. Among them blockage and infection.
• This paper presents a case of 44 years old man admitted to our hospital after an obstruction of his ventriculo-peritoneal shunt by an air bubble that caused behavioral problems and confusion. The patient was operated twice, the last time the puncture point had to be changed.
• Shunt malfunction continues to be a common neurosurgical problem in patients with shunted hydrocephalus, often leading to frequent and sometimes lengthy hospital stays. This case illustrates the management of this rare situation causing air bubble shunt obstruction.

## 1 Introduction

Ventriculoperitoneal shunt (VPS) placement is one of the most commonly performed neurosurgical procedures and is necessary to treat most forms of hydrocephalus [1]. Unfortunately, complications related to VPS placement are common, and multiple. Among them blockage and infection. Early and prompt detection of shunt dysfunction is vital as delay can lead to markedly raised intracranial pressure, coning and death. shunt revisions are almost expected throughout a patient's lifetime [2]. Here we report a case of 44 years old man admitted to our hospital after an obstruction of his ventriculoperitoneal shunt by an air bubble that caused behavioral problems and confusion. the purpose of this case is to demonstrate the superiority of the occipital horn puncture instead of the frontal horn puncture in shunt failures due to pneumocephalus.

## 2 CASE report

A 44-year-old man, right handed, presented in consultation with a heavy neurosurgery past. His history of his disease goes back to 20 years ago when the patient presented a nasal flow by his right nostril similar of a rock water liquid. The biologie revealed that was cerebrospinal fluid, which he decided to consult and MRI was made revealing an important triventricular hydrocephalus on stenosis of the aqueduct of sylvius due to a compression of a tumor with LCS fistulas in the right ethmoidal sinus.

Therefore, it was decided for the patient to undergo a ventriculo-peritoneal shunt for his hydrocephalus and then close the fistulas by a frontal approach and a clinical and radiological monitoring of the tumor given its benign mature and non-voluminous.

The patient had a meningitis complication during his stay and benefited an external shunt, but he came out of it at the end of the hospitalization.

He kept a slight pneumocephalus without clinical effect and he was regularly viewed in consultation.

**Fig. 1:**
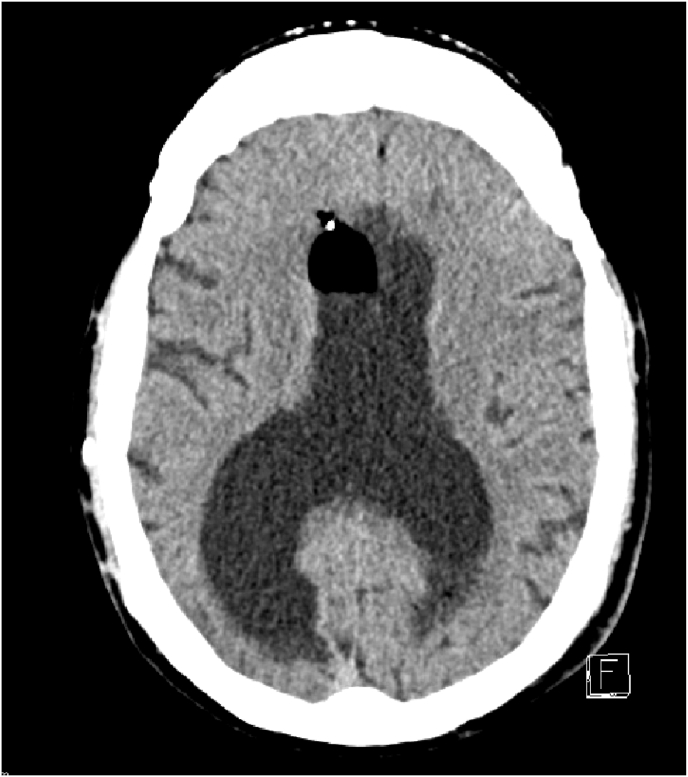
CT SCAN: hydrocephalus with pneumocephalus and pneumoventricle.

**Fig. 2:**
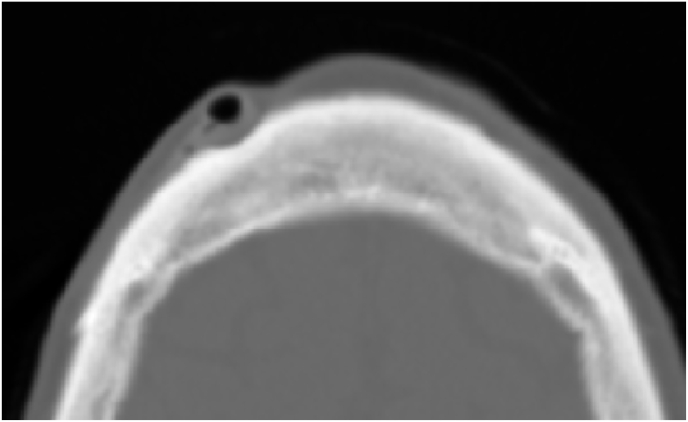
CT SCAN: bublle air in the shunt chamber.

Until a few days ago when the patient presented visual disorders with behavioral problems and rapidly progressive confusion on the cerebral CT scan we found a pneumocephalus with pneumoventricle and a ventricular dilatation Fig. 1, Fig. 2 clinically he was without motor or sensory deficit but he has a walking disorders and disorientation in space and time with 13/15 in his Glasgow score. Ophthalmologic examination was normal and he hasn't a nasal flow.

**Fig. 3:**
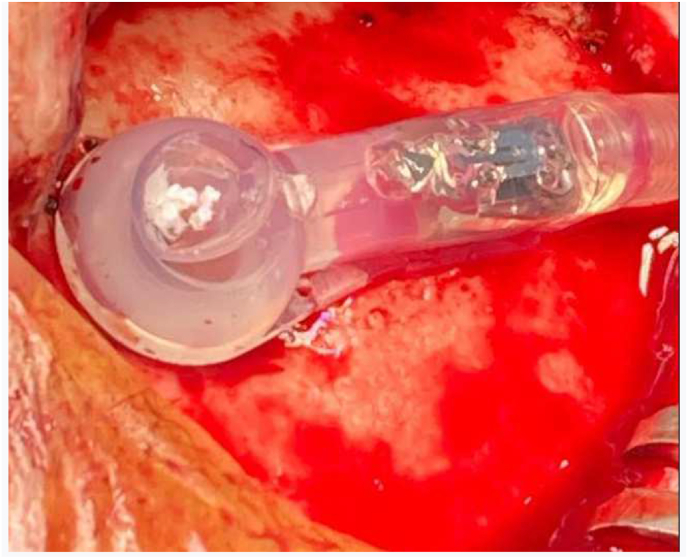
Per operative bublle air in the shunt chamber.

We operated the patient it was made by our head chief department. We made a revision of the shunt on per operative we found an air bubble inside the chamber so we decided to change the all system Fig. 3.

**Fig. 4:**
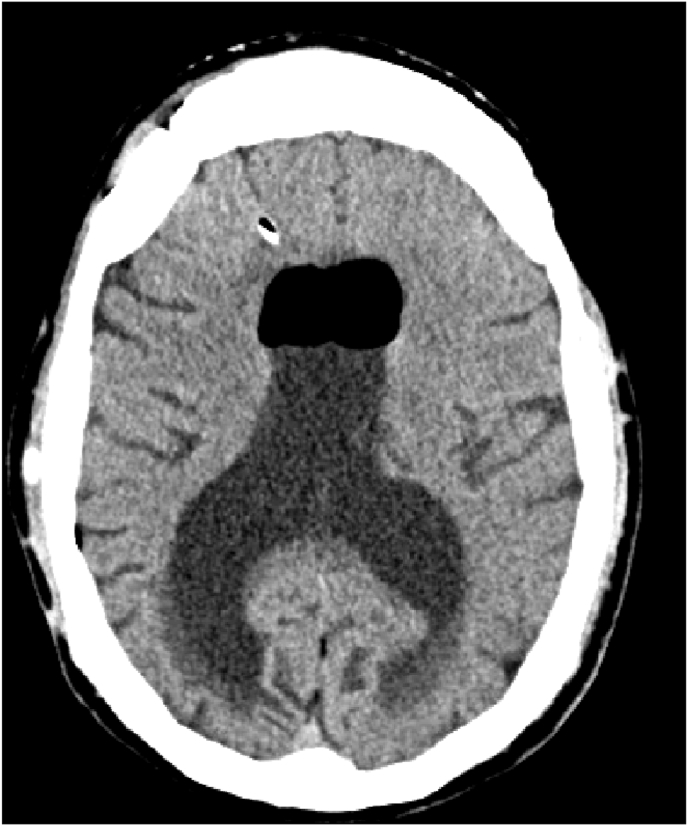
CT scan revealed the persistence of hydrocephalus and pnemocephalus

**Fig. 5:**
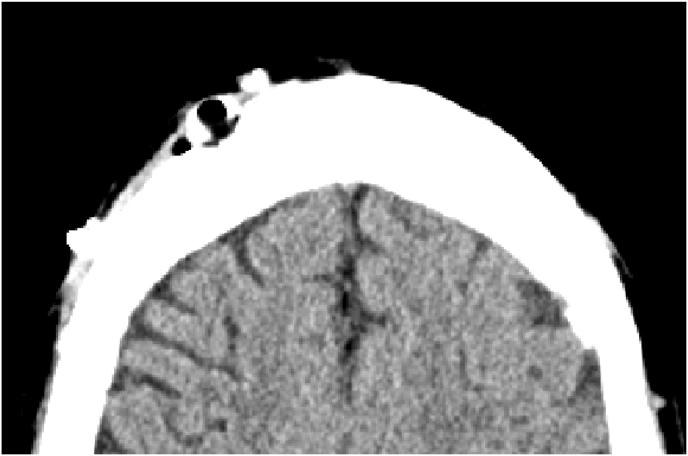
CT scan revealed the persistence of the air bubble.

The follow up was not good no improvement the control CT scan revealed the persistence of the same lesions with the presence of the air bubble in the chamber again Fig. 4, Fig. 5.

We decide to operate a second time. We remove this time the shunt and we placed in the right occipital ventricular puncture instead of frontal one.

**Fig. 6:**
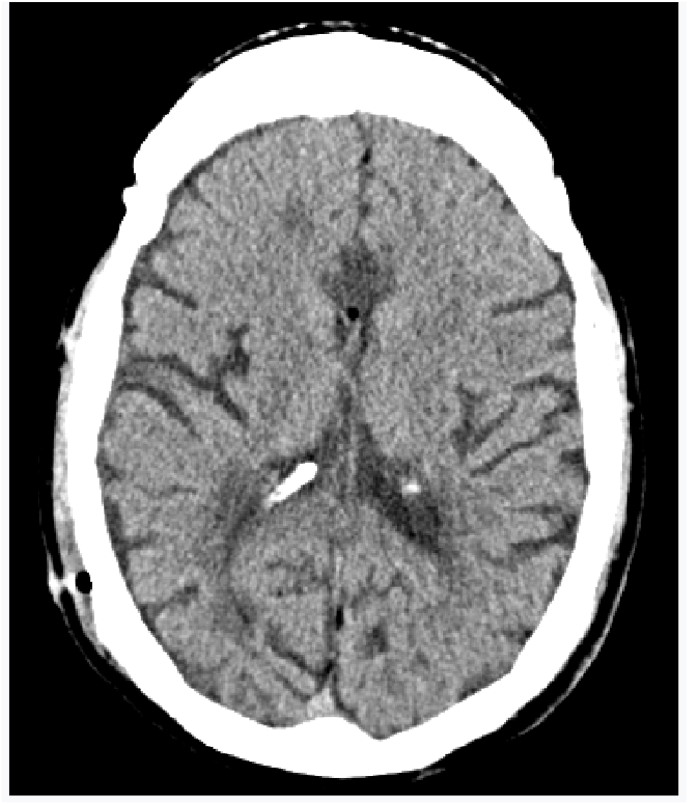
Follow op CT scan next day revealed the regression of hydrocephalus.

The follow op was good total recovery the symptoms disappeared the control CT Scan showed normal ventricles with a slight pneumocephalus Fig. 6.

**Fig. 7:**
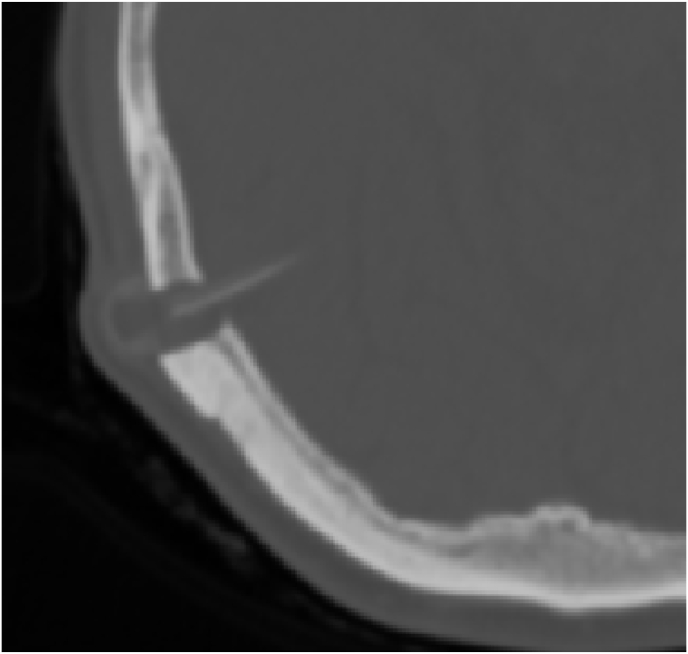
Control CT scan 1 month later revealed the regression of the bubble air.

**Fig. 8:**
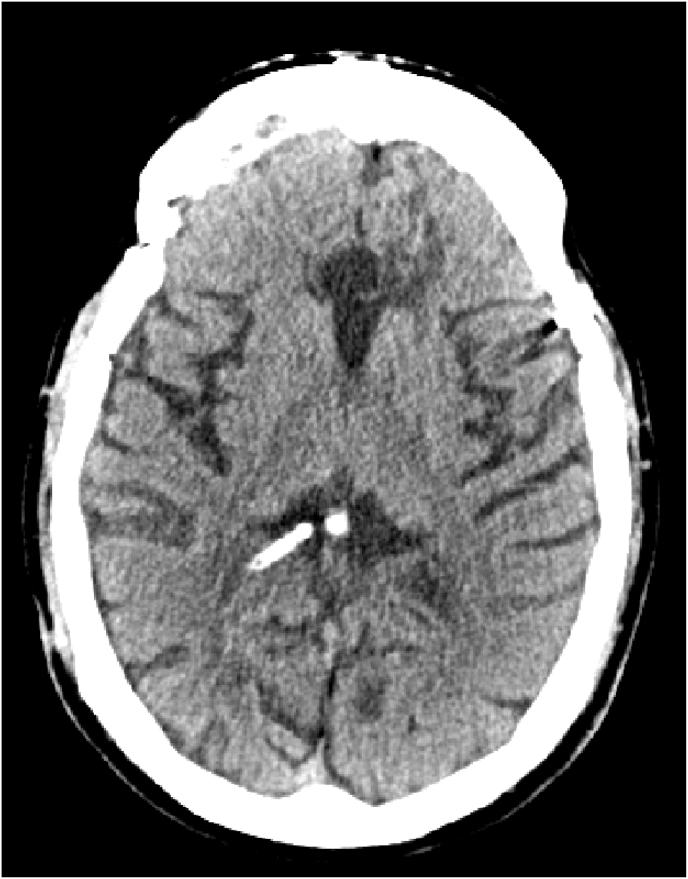
Control CT scan 1 month.

The patient was discharged from the hospital 5 days after the last operation, we saw him again in consultation 1 month later, without any particularity, without complaint, normal neurological examination and ct scan revealed a good ventricular system and disappearance of the pneumocephalus Fig. 7, Fig. 8.

This case has been reported in line with the 2020 SCARE guidelines [3].

## 3 Discussion

Ventriculorperitoneal shunt (VPS) placement is a common neurosurgical procedure with approximately 30,000 shunt procedures performed annually in the United States, however, complication rates remain considerably high. VPS failure rates have been estimated at approximately 11–25% within the first year after initial shunt placement, with most sources reporting a significantly higher number of shunt revisions and replacements among pediatric patients compared to adults [1].

Shunt catheter obstruction is by far the most common cause shunt malfunction, yet the factors that contribute to this problem remain elusive. Obstruction can occur in the proximal catheter, within the valve, or within the distal catheter, however, the most common site of obstruction sited in most studies is the proximal catheter. Multiple studies have compared shunt failure rates between patients having different types of shunt valves, including programmable and non-programmable valves, however, the majority of studies have failed to demonstrate such an association [1].

The introduction of air into components of a ventriculoPeritoneal Shunt (VPS) is a recognized cause of shunt obstruction and failure. Poor ventricular compliance potentiates pneumocephalus when ventricles fail to collapse at lower intracranial pressure and upon exposure to the atmosphere [5]. Surgery has been reported to reestablish the VPS fluid column to permit adequate Cerebrospinal Fluid (CSF) drainage [5]. Reconstitution of CSF flow, however, may also be achieved by repositioning the patient to mobilize the intra-ventricular air pocket away from the ventricular catheter tip [4].

Iatrogenic pneumocephalus is common, although it is usually benign and typically does not require treatment. One study documented that intracranial air is present in all after opening the pseudomeningocele. Theoretically, the influx of air into the intracranial cavity is greater in the presence of a noncompliant ventricular system because the ventricles do not collapse as the fluid is drained and more air is allowed to fill the ventricle [5].

There is also risk of seizure and rapid neurological deterioration due to tension pneumocephalus. Once this occurs, close monitoring of the patient, rapid and accurate identification of tension pneumocephalus, and immediate surgical intervention is life-saving. Gore et al. have advocated the use of 100% oxygen for rapid resolution of pneumocephalus [6].

A patient had pneumocephalus during an ICP placement in Department of Neurosurgery, St. Barnabas Medical Center, USA. To Restore the CSF column within the VPS system was established without surgery by mobilizing patient to the prone position thereby allowing the air bubble to ascend to the occipital horn. CSF re-entered the shunt components by manually pumping the valve approximately 10 times. Upon moving patient back to the supine position, a substantial volume of intra-ventricular air migrated to the temporal horn allowing the catheter to remain in CSF. This was documented by serial bedside lateral skull radiographs. Within approximately 20 minutes of the onset of symptoms the patient improved significantly and imaging the following day demonstrated normal ventricular dimension [4].

In our case we opted for a change of entry point and ventricular location punctured given that the pneumocephaly is found in particular in the frontal horn due to the mechanical law allowing a rapid improvement of the patient and then a gradual elimination of the pneumocephaly.

In other study of Neurosurgery, Icahn School of Medicine at Mount Sinai, New York. They present a pneumoventricle secondary to an iatrogenic cutaneous-ventricular fistula after placement of an external ventricular drain. A surgical technique to manage this condition by replacing air with saline in a non-traumatic manner by placement an external ventricular drain [7]. But in this study the pneomocephaly was important taking all the ventricular system while ours was a minimal quantity of air but it was the migration of the air bubble obstructing the system of shunt which poses problem.

## 4 Conclusion

Shunt malfunction continues to be a common neurosurgical problem in patients with shunted hydrocephalus, often leading to frequent and sometimes lengthy hospital stays. This case illustrates the management of this rare situation causing air bubble shunt obstruction.

## Ethical approval

Written informed consent for publication of their clinical details and/or clinical images was obtained from the patient.

Ethical approval has been exempted by our institution.

## Sources of funding

None.

## Author contribution

Marouane MAKHCHOUNE: Corresponding author and writing the paper.

Michel TRIFFAUX: writing the paper.

Triantafyllos Bouras: writing the paper.

Xavier HAMOIR: Correcting the paper.

## Research registration number

None.

## Guarantor

MAKHCHOUNE MAROUANE.

## Provenance and peer review

Provenance and peer review.

Not commissioned, externally peer-reviewed.

## Declaration of competing interest

The authors declare having no conflicts of interest for this article.
